# Do animal health models meet the needs of organic and conventional dairy farmers in Spain and the UK on disease prevention?

**DOI:** 10.1016/j.vas.2021.100226

**Published:** 2021-12-23

**Authors:** Isabel Blanco-Penedo, Ruth Wonfor, Richard P. Kipling

**Affiliations:** a Animal Welfare Subprogram, IRTA, Veinat de Sies s/n, Monells, Girona 17121, Spain; b Dept. of Clinical Sciences, Unit of Veterinary Epidemiology, SLU, Swedish University of Agricultural Sciences, Uppsala, Sweden; c Institute of Biological, Environmental and Rural Sciences (IBERS), Aberystwyth University, Aberystwyth, Ceredigion, SY23 3DA, United Kingdom

**Keywords:** Dairy farms, Decision support tools, Livestock disease prevention, Farm management, Organic farming, Stakeholder engagement

## Abstract

Modelling plays an important role in assessing disease risks and the efficacy of preventative actions. However, the extent to which existing models meet the needs of different groups of dairy farmers around disease prevention is unclear. A questionnaire gathered information on disease prevention actions undertaken by organic and conventional dairy farmers in Spain and the UK, and on their information preferences and needs in relation to such actions. A systematic review of animal health modelling articles was undertaken to compare the expressed needs of dairy farmers for information on disease prevention, with the focus and outputs of existing models. Farmer groups differed in needs when planning disease prevention interventions. Most farmers sourced animal health information from veterinarians. Farmers preferred to use practical experience to judge the efficacy of change. To fulfil the expressed needs of dairy farmers, models need to address specific farming contexts and non-economic impacts of change.

## Highlights

• Animal health info needs of organic vs conventional dairying in two nations compared.
• Farmer groups differed in needs when planning disease prevention interventions.
• Most farmers sourced animal health information from vets.
• Animal welfare impacts are not well represented in modelling of disease prevention.
• Models need to address specific farming contexts and non-economic impacts of change.

## 1 Introduction

Livestock production systems are currently facing multi-faceted challenges which require them to improve their sustainability, environmentally and economically (Augustin, Udabage, Juliano & Clarke, 2013). High standards of animal health and welfare are a vital aspect of improving food quality, reducing antibiotic use on farms, improving production efficiency and reducing greenhouse gas emissions, and are key dimensions of the European Commission's “Farm to Fork” strategy (EC, 2020). Through their management choices, farmers play a decisive role in raising animal welfare and health standards on their farms, essential to effectively reducing disease incidence and spread. Therefore, it is important to ensure that dairy farmers have access to the support they require to minimize on-farm livestock disease risks (Emanuelson, Sjöström & Fall, 2018).

Farmers’ decision-making processes are based on a complex range of factors, including intrinsic motivations, moral convictions, social preferences, reciprocity, and the impact of peer groups (de Lauwere, van Asseldonk, Bergevoet & Bondt, 2020) in addition to economic imperatives. Farmers are more likely to adopt innovation and change if they expect it to help them achieve their aims, which may include economic, social, environmental (Greiner, Patterson & Miller, 2009), and animal welfare goals (Liu, Toma, Barnes & Stott, 2019). However, challenges to change in the form of practical (available resources, nature of existing system), knowledge (of options for change, effective implementation and likely outcomes) and cognitive (strategies to manage complex information and conflicting aims under pressure) barriers may prevent change even where it aligns with the farmer's own perceived interests (Kipling, Taft, Chadwick, Styles & Moorby, 2019). As such, requirements for resources to support change are likely to vary between farming systems and locations as well as between individual farmers (Brodt, Six, Feenstra, Ingels & Campbell, 2011).

Mathematical models and decision support tools can play an important role in addressing gaps in knowledge amongst farmers and in untangling the complex factors affecting animal health in order to improve decision making (Özkan et al., 2016). Models must be seen as credible, legitimate and relevant by stakeholders in order to drive change (van Bruggen, Igor & Kwakkel, 2019). amongst other things this requires a focus on the communication of modelling to stakeholders (Kipling & Özkan, 2016), acknowledging the importance of how, when, in what form and by whom knowledge is provided (Pothmann, Nechanitzky, Sturmlechner & Drillich, 2014; Sjöström et al., 2019; Svensson et al., 2020).

Many model and support tools for risk mitigation is available for risk managers on animal health. Improving the extent to which such tools incorporate the needs and perspectives of farmers both in their scope and outputs would represent a crucial step forward for improving the uptake of effective disease prevention measures. At the same time, better understanding of differences between the needs of different groups of farmers can inform improvements in the tailoring of the whole range of knowledge exchange approaches used in the farming sector to address specific issues in specific contexts (Kipling & Becoña, 2019).

The nature of dairy farming systems and the health challenges they face differ between European countries (EFSA, 2009, 2012). Such differences are exemplified by Spain in southern Europe, and the UK in northern Europe. In Spain, dairy herds are on average smaller (Blanco-Penedo et al., 2019; MAPAMA, 2020) than in the UK, are more likely to be housed or kept in open yards all year round in contrast to a greater reliance on grazing in the UK (AHDB, 2021) and are likely to be affected by climate change in different ways, with heat stress an ever-increasing issue in southern Europe (Blanco-Penedo, Velarde, Kipling & Ruete, 2020) and issues relating to warmer, wetter winters and associated disease impacts challenging UK farmers, such as helminth burdens (Fox, Marion, Davidson, White & Hutchings, 2012; van Dijk, Sargison, Kenyon & Skuce, 2009). In this context, it is important that the decision support around animal health provides resources tailored to the differing needs of contrasting geographical and socio-economic conditions (Perry, Robinson & Grace, 2018). Although animal health advice needs are likely to differ between conventional and organic systems (Krieger et al., 2020; Sutherland, Webster & Sutherland, 2013; Vaarst et al., 2008) few models have focused on the specific needs of organic dairy systems (James, Antle & Wheeler, 2017). .

There is wide variation between European countries in the current share of dairy farms which are organic, and although in the EU organic milk production has increased almost two-fold, it still only constituted 3.4% of overall EU milk production in 2019 (Willer, Trávníček, Meier & Schlatter, 2021). Farm conversions have taken place in response to the dairy crisis, as organic milk can be sold at a higher price than non-organic, and many consumers are turning towards organic products (EPRS, 2018). The EC have proposed the ambitious goal of increasing the share of organic agriculture to 25% by 2030 (EC, 2020).

Current European Union Animal Health Law (Regulation (EU) 2016/429), states that farm biosecurity is a requirement for the effective management of animal health. Such management must be flexible and adaptable to different production systems and local contexts (Moya et al., 2020). As such, it is vital to understand the extent to which the needs of organic and conventional dairy farmers differ in relation to disease prevention, and whether these different groups have access to the support they need to minimise health risks to their animals.

Spain and the UK have contrasting dairy cow production sectors, providing an opportunity to explore differences in the needs of farmers across systems and countries in relation to decision support for disease prevention. The proportion of organic dairy cows to conventional dairy cows in the UK and Spain in 2020 was similar, at 1.9% and 1.4% respectively (DEFRA 2021; EUROSTAT, 2021). In 2018, an estimated 80–95% of dairy cows in the UK were grazed for at least part of the year versus only 20% in north west Spain, reflecting climate-related differences in the availability of grass (van den Pol-van Dasselaar, Hennessy & Isselstein, 2020). All farms in the UK pay a statutory levy board fee and are members of the Agriculture and Horticulture Development Board (AHDB) which provides advice and support on good practice. In Spain, organic dairy farming is largely constituted by small-scale family farms with a low participation to cooperative dairy companies and low access to expertise (Blanco-Penedo et al., 2019). There is a heterogeneity in veterinarian profiles in all dairy cattle farms (e.g. private veterinarians, veterinarians involved in animal health programs-either in an animal health defence associations (HDA) or a public company official veterinary services). This might generate different perceptions and recommendations to farmers (Moya et al., 2019).

The aims of this study were to 1) assess the extent to which the information priorities, sources, uses, and needs of dairy farmers differ between two European countries (Spain and UK) and between organic and conventional farming systems and, 2) to assess the extent to which current animal health models in the scientific literature meet the information requirements of dairy farmers around disease prevention, including differences in needs between country and system.

## 2 Materials and methods

### 2.1 Farmer questionnaire

A questionnaire was developed by researchers to identify the information priorities, sources, uses, and needs of organic and conventional dairy farmers in Spain and the UK, around on-farm changes to reduce the risk of disease (Table 1; Supplementary Material 1). The questionnaire included tick box questions relating to the information farmers collect on-farm to inform their management, and their preferred information sources when making changes relating to animal health. Farmers were asked to define the most recent change they had made on-farm to reduce disease risk, to ensure that subsequent answers were based on concrete examples likely to focus their responses (Krieger et al., 2020). It was specified that changes should have been a matter of choice, not something required by regulations. The questionnaire then asked about the issues the farmer considered when making the choice to implement a change, the issues they felt they needed more information on, and their aims in making the change. Six types of issue were presented for respondents to select from: practical; economic; animal welfare; risk of not taking action; wider concerns or ‘another reason’ (with a free text box for details). Participants could select as many of these issues as they liked. They were also asked to consider how they evaluated whether a change made to prevent disease was effective. Basic farm structural information was collected, including farm type, breed, husbandry practices, grazing management, and health management.

**Table 1:** Summary of questionnaire distributed between November 2016 and July 2019 to dairy farmers in Spain and UK.

Question no.	Question	Answer type
1	Farm country	Free text
2	Main breed	Free text
3	Days grazed	Tick box
4	Organic or conventional	Tick box
5	Data collected on-farm	Tick box and free text
6	Information sources used by farmers	Tick box and free text
7	Biggest change in last 12 months to reduce disease risk	Free text
8	How thought change would reduce disease risk	Free text
9	Topics considered when making change	Tick box and free text
10	Details of considerations in Q9	Free text
11	Information that would have been useful when making change	Tick box and free text
12	Details of information in Q11	Free text
13	Checks made to see if change is effective	Tick box and free text
14	Information that would have been useful when deciding if change is effective	Free text

Spanish and English language versions of the survey were uploaded at Typeform™ Survey Maker (Typeform©) and distributed in Spain and the UK, using a randomized stratified sampling procedure, between November 2016 and July 2019. In addition, to avoid any bias introduced by limitations in internet access for some farmers, questionnaires were either emailed to farmers through existing contacts with researchers or distributed via a range of dairy associations and dairy farming organizations using social media or members’ publications. In Spain, organic council organizations distributed the questionnaire via email and were also distributed in a seminar organised by one Organic Council in NW Spain. English questionnaires were distributed in the UK at agricultural shows (The Royal Welsh Show 2018 and 2019) and knowledge exchange events (health related farmer meetings organised by AHDB Dairy in 2018).

Quantitative data were analysed:

i To compare the distribution of responses across countries and systems, Fisher's Exact Test was used for the following comparisons –; Spanish organic (ORGSP) vs Spanish conventional (CONVSP); CONVSP vs UK conventional (CONVUK). Due to a low sample number, responses from UK organic farmers were not included in the comparisons.
ii To compare differences in the proportion of responses for each option where multiple options were presented in a question, analysis was undertaken using Chi-square goodness of fit tests with a null hypothesis of equal preference for each response option, applying William's correction for small sample sizes where necessary (Williams, 1993). Where no significant difference was found in the initial country/system comparisons (analysis i), the Chi-squared test was completed on the full sample of responses. However, where the initial comparison (analysis i) found a significant difference, the Chi squared test was completed on the individual country and systems groups of farmers (ORGSP, CONVSP, ORGUK, CONVUK), to understand if proportions of responses were different between groups.

All statistical analyses were performed using STATA (Stata Corp., College Station, TX, USA). Qualitative data in the form of details given when farmers responded ‘other’ to multiple choice questions were used descriptively, to add further insight to quantitative analysis of the relevant question, as were responses to specific questions asking for details relating to the preceding multiple-choice question. Qualitative data from open text responses were explored using thematic analysis, in which responses are coded to identify topics, with coded material sorted into themes and categories to shed light on the research question (Ritchie, Lewis, McNaughton Nicholls & Ormston, 2013).

### 2.2 Assessing model alignment to farmer needs

**Fig. 1:**
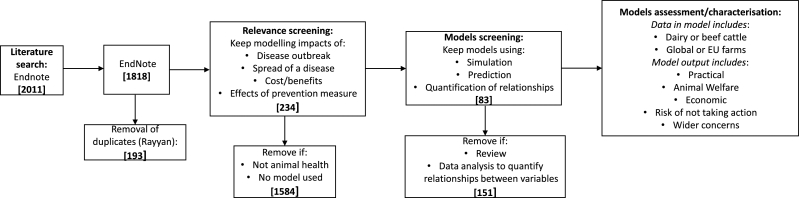
Workflow of the systematic review process. Numbers in brackets indicate the number of journal articles that were either removed or kept from each stage of the review process. The final model assessment and characterisation step was completed on the 62 articles found to be relevant and useable.

A systematic review of studies in peer-reviewed scientific journals was undertaken, to identify modelling studies which included the assessment of disease control measures and/or the impacts of health conditions. An initial search of the peer-reviewed literature was carried out on the 13th of April and the 13th of May 2020 in the Pubmed database according to PRISMA guidelines (Liberati et al., 2009). Search terms were defined using the PICO approach (population, intervention, comparison, and outcome) (Methley, Campbell, Chew-Graham, McNally & Cheraghi-Sohi, 2014). The final search terms (Supplementary Table 2) were classified as: Population – the animal species and types; Intervention – nature of the farming system; Comparison – different disease statuses examined by models; Outcome – impacts on production, health or environment. Search results were then filtered following a stepwise process (Fig. 1). Remaining models were assessed relative to farmer needs revealed by the questionnaire, centring on their focus, scope, and types of output.

Narratives summarizing the qualitative data from the questionnaire were combined with the findings of the quantitative analysis of multiple-choice questionnaire responses, to create descriptions of the needs of different groups of farmers (ORGSP; CONVSP; ORGUK; CONVUK) for each aspect of implementation and evaluation gathered. These summaries were then compared with findings from the systematic review, to assess the extent to which models met the expressed needs of farmers in terms of the disease prevention changes focused upon and the extent to which outputs addressed different types of issue (economic; animal welfare; risk of not taking action; wider concerns).

## 3 Results and discussion

### 3.1 Farmer questionnaire – comparisons between systems and countries

A total of 27 (56.2%) of Spanish farmers in the sample were organic and 21 (43.8%) were farmers from conventional farms. For the UK, 5 (10.4%) were organic and 43 (89.6%) were conventional farmers. As the UK sample did not include enough organic farmers to test for differences between systems, within-country comparisons between conventional and organic systems were only undertaken for Spain.

Comparing the data for conventional farms across both countries (Table 2) revealed that farmers in Spain and the UK considered different factors as part of the change that they made (Question9), with CONVUK more likely to consider routine and CONVSP more likely to consider animal comfort and expense/productivity. No significant differences were found in responses to other questions.

**Table 2:** Comparisons of responses to questions between dairy farmers in different countries and systems. CONVUK = Conventional UK, CONVSP = Conventional Spanish, ORGSP = Organic Spanish. Significant differences between countries/systems indicated by bold P values.

Question	Comparison	Milk records	Breeding records	Farm Book (deaths, culling)			Fisher's Exact test P value
Q5. Types of health information collected on farm	CONVUK	40	40	43			0.847
	CONVSP	18	21	18			
	ORGSP	19	23	16			0.918
	CONVSP	18	21	18			
		Talking to vet	Farming magazines	Searching internet	Farm advisors / consultants	Other farmers	
Q6. Sources of animal health and disease risk information	CONVUK	40	36	18	26	23	0.722
	CONVSP	21	16	15	16	14	
	ORGSP	24	9	3	14	9	0.092
	CONVSP	21	16	15	16	14	
		Practical	Animal Welfare	Economic	Risk of no action	Wider concerns	
Q9. Factors considered when making change	CONVUK	30	8	5	7	2	**<0.0001**
	CONVSP	3	13	16	4	4	
	ORGSP	11	19	16	4	7	0.459
	CONVSP	3	13	16	4	4	
		Practical	Animal Welfare	Economic	Risk of no action	Wider concerns	
Q11. Factors farmer needed to know more about when making change	CONVUK	9	7	13	7	3	0.578
	CONVSP	5	3	10	5	6	
	ORGSP	5	9	4	3	0	**0.024**
	CONVSP	5	3	10	5	6	
		Check monitoring data	Info from farm checks	Trial for a while	Judge based on experience		
Q13. What did farmer do to see if change was effective?	CONVUK	19	8	8	33		0.091
	CONVSP	9	5	11	11		
	ORGSP	9	8	10	16		0.787
	CONVSP	9	5	11	11		

Comparisons of CONVSP and ORGSP farmer responses (Table 2) demonstrated a significant difference in the further information needed when making a change (Q11), with ORGSP farmers requiring further information around ‘wider concerns’ compared to CONVSP. No significant differences were found in responses to other questions.

### 3.2 Farmer questionnaire – preferences within systems and countries

In relation to the types of health information kept on farms (Q5), analysis across the whole sample of respondents (Table 3) rejected the null hypothesis of equal preference for each option in the types of data farmers reported collecting, with most respondents gathering data in all three categories. Other types of information collected at the farm were: data on specific disease incidents; veterinary prescription; non-health and non-animal parameters such as economics or land held (ORGSP / CONVUK); young stock performance and body condition (ORGSP); keeping a diary; using a computer program to monitor pregnancy rates; and milk test (ORGUK). The results indicate that farmers from both countries and farming systems collect a range of health data of potential value for modelling. Further exploration of these resources, and how they might be made more accessible to researchers, would be valuable, given that data availability and quality have been previously identified as challenges for animal health modelling(Kipling et al., 2021) .

**Table 3:** Results of Chi-square Goodness-of-fit tests for within-group differences in preference for question options. For questions where there were no significant differences in system/country comparisons, the whole sample was tested. For questions where system/country comparisons revealed significant differences, individual sample groups (CONVUK, CONVSP, ORGSP) were tested (CONV = Conventional systems; ORG = Organic systems; SP = Spain; UK = United Kingdom).

Question	**Comparison**	**Milk records**	**Breeding records**	**Farm Book (deaths, culling)**			**χ2**	Chi-square Goodness-of-fit P value
Q5. Types of health information collected on farm	Whole sample	77	84	77			0.41	**0.814**
		Talking to vet	Farming magazines	Searching internet	Farm advisors / consultants	Other farmers		
Q6. Sources of animal health and disease risk information	Whole sample	85	61	36	56	46	23.99	**<0.001**
		Practical	Animal Welfare	Economic	Risk of no action	Wider concerns		
Q9. Factors considered when making change	CONVUK	30	8	5	7	2	47.28	**<0.001**
	CONVSP	3	13	16	4	4	17.8	**0.001**
	ORGSP	11	19	16	4	7	13.21	**0.01**
		Practical	Animal Welfare	Economic	Risk of no action	Wider concerns		
Q11. Factors farmer needed to know more about when making change	CONVUK	9	7	13	7	3	6.6	**0.159**
	CONVSP	5	3	10	5	6	4.47	**0.347**
	ORGSP	5	9	4	3	0	9.73	**0.045**
		Check monitoring data	Info from farm checks	Trial for a while	Judge based on experience			
Q13. What did farmer do to see if change was effective?	Whole sample	37	21	29	60		23.1	**<0.001**

A whole sample Chi-square goodness-of-fit test found that there was a significant difference in preference for the different sources of information used by farmers (Q6) versus a null hypothesis of equal preference for each option (Table 3a). The most farmers reported talking to their veterinarian and the fewest searching the internet for information. In the details provided in relation to this question, CONVUK farmers reported gaining information from farm visits and contacts with levy boards and the farm advisory service. These sources could be classified broadly as gaining information from farm advisors. No further details were provided by ORGSP nor by CONVSP farmers. Consistent with the findings on sources of information used by farmers reported here, previous research has highlighted low computer literacy amongst farmers and connectivity issues in rural areas (International Telecommunication Union & FAO, 2020). Our findings and previous work therefore suggest that modellers (and others seeking to provide information and advice on animal health) should be cautious about using online resources to get their messages across to farmers directly. Although veterinarians may be able to access and pass on such information to farmers, direct communication is also important, empowering farmers to make informed choices and enabling them to identify issues and solutions to ask for advice on.

In organic farming, previous studies have identified weaknesses in current farm advisory provisions on health, with veterinarians sometimes providing the only support for farmers on this topic (Blanco-Penedo et al., 2019; Duval et al., 2017). In the UK, some agricultural stakeholders feel that veterinarians could and should play a bigger role in providing advice on disease prevention and control, beyond administering treatments (Shortall et al., 2016; Kipling et al., 2019). Other studies support the view that the advocacy of veterinarians for preventive measures has been limited and could be improved through better communication (FVE, 2021; OIE, 2017), including the provision of more tailored, context specific advice by veterinarians to improve relationships with farmers (Krieger et al., 2020).

Within each group of farmers there were significant differences from the null hypothesis of equal preference for topics considered when making changes (Q9) (Table 3). For ORGSP farmers, animal welfare as well as the economics of changes were the focus, while the least mentioned factor considered was the risk of no change. Details given about these responses suggested that for some ORGSP farmers the importance of health and welfare was connected to performance improvements or reduced workload “*The improvement of the bedding influences a lot in the animal welfare and therefore in the economy*”. This understanding may explain why relatively few of the ORGSP group reported considering wider impacts, economic consequences of outbreaks or the impact of new measures on routine as separate issues. Concern about these factors may be implicit in concerns for their animals, as already reported by Alrøe, Vaarst and Kristensen (2001).

The highest proportion of CONVSP farmers focused on the economic impacts of change, with animal welfare the second most mentioned topic, while there were few responses indicating consideration of the practical impacts of change, risks of no action, or wider concerns (Table 3). CONVUK respondents differed from their Spanish counterparts in that the biggest proportion of responses was for considering practical impacts of change; again, few responses indicated consideration of wider concerns such as alternative options/environmental costs and benefits of the change (Table 3). Notable in the details given about these factors was that several respondents were keen to reduce their reliance on antibiotics, this may reflect recent concern about antibiotic resistance in the literature (Krogh, Nielsen & Sørensen, 2020) and farmers’ knowledge of best practice, perceptions and experience of the issue (Fischer, Sjöström, Stiernström & Emanuelson, 2019). No details were given by three respondents (across the whole sample) who reported considering factors beyond those covered by the categories in the multiple-choice part of the question.

In relation to topics on which farmers would have liked more information when making a change (Q11), the proportions of responses in each category did not differ significantly from the null hypothesis of equal preferences for each option for CONVSP or for CONVUK (Table 3), although in both cases the biggest proportion of responses was for information on the economic impacts of change. Details provided by respondents showed that some CONVSP farmers wanted more information on the economic costs of the action, including if it failed. One was interested in the response of animals to the action, and another would have liked information on the efficacy of the disinfectant product being applied, with a guarantee. amongst ORGSP respondents, the proportion of responses was highest for information on animal welfare, while none reported a need for more information on wider concerns about an action (Table 3). Few ORGSP farmers provided more detail about their responses, although some wanted to know more about the management of the considered change in terms of cost and time and indicated that they would like to benchmark their performance against those of other farmers. Soil analysis, disease management in general, and legislation were also raised as topics of interest, even though no ORGSP respondents reported wanting more data on wider concerns relating to their change.

When farmers do not indicate that they require more information on wider concerns about a change (the questionnaire gave ‘impacts on environment or alternative options’ as examples) this might suggest three things: i) a lack of awareness of wider impacts, ii) farmers feeling they have enough information on wider impacts already, or iii) farmers feeling that wider impacts were not important enough to explore relative to other demands on their time. Comparing the answers to Q11 with answers to Q9(issues considered when making a change), showed that 12% of ORGSP farmers considered wider concerns, but none wanted further information on them, suggesting a fit to explanation (ii), while the responses of the remainder (who did not consider such concerns) fit explanation (i) or (iii). A small number of CONVSP and CONVUK farmers considered wider concerns and the same number of respondents indicated they would like more information on the topic. This suggests such farmers had an interest in wider issues but were not finding the information they needed but, again, that the majority of famers fitted explanation (i) or (iii). To tackle a lack of awareness amongst farmers of the wider impacts of on-farm change and to reflect the importance of such wider concerns to society, information on the economic impacts of changes could be integrated with environmental and social effects when giving advice (Schnyder, Auerswald, Geist & Heissenhuber, 2019).

On the actions taken to see if change was effective (Q13), across the whole sample results rejected the null hypothesis that all types of action would be equally preferred (Table 3). The most reported test of efficacy was judgement by practical experience, with the smallest proportion of respondents citing the use of data from farm checks (audits, etc.). Respondents who used other approaches to evaluation reported referring to a handbook (ORGSP), focusing on time (either stating that change would take time or that they would wait until the action was likely to have made a difference), using their own experience and observations (fitting closely to the practical experience category; CONVSP), using a disease specific plan, or relying on monitoring data (CONVUK).

CONVSP farmers gave no details of further information that would have been useful in evaluating the effectiveness of change (Q14). amongst ORGSP farmers, responses indicated that print outs of important data would be valuable, or focused on how they might use information “*A more professional vision to improve the implementation*”. The latter responses highlight that knowledge and the capacity to process and make choices based upon it are two different challenges, with the potential for increases in information to increase the complexity of choice-making (Kipling et al., 2019). CONVUK farmers provided many ideas on this topic, highlighting the usefulness of practical farm trial data and communication with others who had made a similar change, or with a veterinarian. The cost of information and the availability of products required for changes were also mentioned. Others focused on specific topics around herd performance.

Taking the responses to Q13 and Q14 together reveals strong preferences amongst farmers for experience and judgement (including the experience of others who have already implemented a change or data from trials). Judging from experience may be seen as a way of avoiding being overwhelmed by complex information, which may be challenging to process. Previous research has shown that copying the (successful) example of others can take over from habitual farming practices as challenges arise, with both representing mental short cuts which avoid ‘starting from scratch’ in addressing a problem (Mankad, 2016). Collecting monitoring data can also be characterized as a process of learning from practical experience and may support judgement calls rather than adding complexity to them. In contrast, scientific information and advice about risks or opportunities requires time and mental space to process and use as a basis for action. This cost is increased if information must be paid for or if options for change are not available (Kipling et al., 2019).

The (few) ORGSP responses to Q13 and Q14 suggested a need to improve the usability of data and the support available to process and come to decisions based upon it. Along with responses to Q13, the contrast between the range of answers from CONVUK farmers and the lack of any responses from CONVSP farmers suggest a lack of a sense of agency amongst the latter group in relation to making changes. In support of this hypothesis, earlier Spanish studies (Esparcia, Mena & Escribano, 2014; Moya et al., 2020) report that there are insufficient mechanisms for transferring new knowledge and innovations to farmers in Spain, and that the information offered is not sufficiently adapted to the farmers’ needs. Further investigation into Spanish farm advisory services is required to explore these issues in more depth.

### 3.3 Farmer questionnaire – analysis of open text responses about changes made

Open text responses to the question ‘What was the biggest change you made on your farm in the last twelve months that you hoped would reduce disease risk?’ (Q7) grouped into four themes, relating to: 1) the animals “*we changed to PROCROSS breed since they are more resistant to metabolic and infectious diseases*”, 2) changes in infrastructure “*fans to increase airflow and reduce pneumonia*”, 3) changes in management “*calf rearing - clear pens and colostrum within 1 to 2* *h of birth*” or 4) changes in the application of disinfectant products to reduce disease risks “*Disinfection of bedding and alleys, milking room*”. For ORGSP respondents, the focus was most often on changes around stalls or bedding material for animals, centring on actions relating to hygiene and involving infrastructure, management or products (e.g., vaccination, disinfectants, and cleaning products). Altering the timing of housing and grazing were mentioned by some (management actions) and vaccination was also reported. All four action themes were represented amongst CONVSP respondents, including alterations to personnel as an aspect of management change “*Bad formulation of the TMR* [Total Managed Ration] *– we changed the consultation nutritionist*”, changing cattle breed (Animal related action) and again the use of new products, such as new bedding material or vaccines. Product (including disinfectant, vaccines, and testing) and management-based changes (cleaning practices including around stalls and bedding, using/supplementing colostrum for calves, caution around buying new stock) as well as infrastructure changes (e.g., to sheds and stalls, for ventilation and space) were reported by CONVUK respondents relating to a range of aspects including hygiene (in stalls and bedding and more widely).

In terms of the mechanism by which farmers expected their actions to reduce disease risk, many respondents just gave details of the specific health condition they were aiming to address, misunderstanding the question posed – a range of diseases were mentioned including mastitis (and reducing somatic cell count) and, claw health/lameness. Beyond these types of response, ORGSP farmers focused on reducing infection risks, mentioning reducing exposure of animals (on-farm or off-farm) to pathogens or poor conditions. These responses were to be expected because of a greater reliance on prevention than treatment in organic systems, associated with an avoidance of the blanket use of antibiotics as a preventative intervention (EC 889/2008; Krogh et al., 2020).

CONVSP respondents commented on their level of expectation about whether their action would work, including having no expectations but being tired of using antibiotics. Although the questionnaire asked respondents to focus on non-mandatory changes, one farmer still reported acting because “*I didn't believe in advisors but when I needed to cull animals I didn´t have other options*” while another did not trust his/her advisor and so chose not to act. CONVUK farmers cited expectations of improving immunity or resistance to disease in their herd, of reducing on- and off-farm exposure to pathogens and improving environmental conditions, and of improving the identification of disease. Previous work around on-farm change suggests that a sense of agency (feeling that one's actions can make a difference) is an important aspect of decision-making (Mankad, 2016). In this context, the negative responses of some CONVSP farmers in relation to their expectations of change are concerning, along with (in comparison to CONVUK farmers) less indication of an understanding of the mechanisms via which changes might have an effect.

The range of disease prevention measures detailed in the open text answers discussed above, and the specific motivations and expectations for change, show great variety and overlap with other management issues, such as the personal relationships between farmers and advisors. These findings highlight the context-specific nature of disease prevention, and farm management in general. The likelihood of success in delivering health prevention improvements is highly dependant on the motivation of farmers to undertake change, and on the availability and quality of farm resources (Vaarst et al., 2008; Bennedsgaard, Klaas & Vaarst, 2010; Ivemeyer et al., 2012; Vaarst et al., 2011).Veterinarians and other health advisors need to understand the structure of their client's farm system and its context (Kristensen & Jakobsen, 2011) in order to appreciate and tackle the barriers to change which are present (Kipling et al., 2019).Providing farmers with rational but universal arguments for change might not always be sufficient to motivate on-farm action (Ritter et al., 2017). Linking farm characteristics to variation in the implementation of health improvement changes (Blanco-Penedo et al., 2019), may be a useful approach in achieving more context-specific and relevant advice and support to farmers.

### 3.4 Assessing model alignment to farmer needs

The data presented above were summarized to produce narratives for each group of farmers (ORGSP, CONVSP, CONVUK) and framed in terms of model requirements (Table 4). The results from Q5 were not relevant for the comparison with model characteristics, representing an investigation of sources of data for modelling, rather than farmer needs. Q6, Q13 and Q14 were also excluded from this part of the analysis, as published articles (the focus of the systematic review) would be unlikely to describe how/if the outputs of the surveyed models were disseminated/presented to farmers, or how the model could be/had been used by farmers in decision making.

**Table 4:** Narratives relating to questionnaire responses for each group of farmers (CONVUK = Conventional farmer in UK, CONVSP = Conventional farmer in Spain, ORGSP = Organic farmer in Spain) framed as model requirements to provide a basis for comparison of farmer needs and existing health model focus and outputs.

Question	CONVUK	CONVSP	ORGSP
Required model focus (Q7 plus specific conditions in Q8 responses)	Focus across a broad range of actions but with emphasis on infrastructure changes in housing, as well as vaccination and testing, controlled addition of new stock, and calf feeding. Focus on a range of health conditions, with mastitis and lameness most mentioned.	Focus across a broad range of actions including changes in products used (e.g. bedding), management, and animal breed. Focus on a range of health conditions, but with mastitis most mentioned.	Focus on management, infrastructure and product use changes particularly relating to stalls and bedding. Include changes to the timing of activities, such as time spent housed. Focus on claw health/lameness and mastitis.
Model capacity to characterise expected pathway to change (Q8)	Characterise processes and consequences of improved identification of health problems, increased immunity and resistance. Modelling of infection rates from the on-farm or off-farm environment. and of changes in levels of disease arising from environmental conditions	Characterising the effects of change across the system would be valuable as this group did not focus on any specific mechanisms of change and instead considered the extent to which they expected success – providing information about mechanisms of change might support more informed and empowered decision-making.	Characterise impacts of proposed changes on infection rates from the on-farm or off-farm environment and characterise impacts of changes in levels of disease arising from environmental conditions. Facilitate benchmarking between farms.
How to engage farmers with findings (Q9, Q10)	Focus on impacts of changes on workload and routine to engage with farmers’ concerns.	Focus on the costs and production impacts of changes and on animal welfare to engage with farmers’ concerns.	Focus on animal welfare and the costs and production impacts of changes to engage with farmers’ concerns.
Model outputs (Q11, Q12)	Focus on economic costs of implementing change, but farmers’ reported preferences did not differ significantly between categories of information they reported lacking – as such, incorporating a wide range of model output types would be beneficial. Modelling of ongoing costs of a change over time and assessment of optimal solutions were additional aspects arising from qualitative answers.	Focus on economic costs of implementing change, but farmers’ reported preferences did not differ significantly between categories of information they reported lacking – as such, incorporating a wide range of model output types would be beneficial. Qualitative responses suggest that modelling of the costs of failure and the efficacy/reliability of products would be valued.	Focus on animal welfare as the information type most requested. For a minority of this group also consider wider impacts of change andcosts of inaction

Of the 62 models assessed following filtering of the literature sample (Supplementary Table 3), the outputs of 40 (64.5%) focused on the economic impacts of change, 13 (20.9%) on the practical impacts of change, 1 (1.6%) on animal welfare impacts, 19 (30.6%) on the risk of inaction, and 7 (11.3%) on wider concerns (such as alternative options or environmental impacts). Individual surveyed models only covered an average of 1.29 of these five output categories. Eleven of the models looked only at the efficacy of changes in the reduction of disease, without consideration of any of their costs or impacts.

The attention paid by modellers to economic impacts of change aligns with the high proportion of responses from CONVSP farmers indicating that they considered this topic when making the decision to implement a disease prevention intervention. However, only 20.9% of models considered the practical impacts of change which were of most interest to CONVUK farmers and, although data from the questionnaire suggest that animal welfare was an important aspect for ORGSP farmers (both in terms of issues considered and additional information that would have been useful), only one model considered animal welfare impacts, suggesting that this concern of farmers is not well represented in modelling. The economic focus of many models implies a perspective that decisions around health should be based solely on financial costs and benefits. This represents a tension between perspective of farming as a business, and farming as a way of life in which good stockmanship is a matter of pride and care rather than simply a financial choice. CONVSP and CONVUK farmers’ preferences for additional information did not vary significantly between categories, suggesting that models covering multiple impacts of intervention would be useful for this group and, as such, addressing the narrow focus of models indicated by the low average number of topics covered could be a focus for future model development.

From qualitative responses, the models considering only the impacts of change on disease rates may be of interest to farmers wanting to know more about the costs of a failed intervention and the reliability of products. Reflecting the multiple-choice responses on this topic, open text data from CONVUK farmers focused on concern with practical impacts of change, covered by 20.9% of models – modellers might therefore address such issues more when aiming to engage with this group.

In terms of disease focus, the 62 dairy-specific modelling articles in the sample (Supplementary material 3.1) mostly considered a single type of health condition, with the largest number (27 or 44% of the sample) focusing on mastitis. Coverage of other health conditions was even. These figures are a good fit with the focus of farmers across all three groups (ORGSP, CONVSP and CONVUK) on mastitis as the condition most targeted by interventions. Four models looked at a range of health conditions beyond the categories defined by thematic analysis, with a further eight considering more than one condition within, or looking in general at, a particular focus category (e.g., fertility). A big challenge for animal health modelling is to move beyond the characterisation of disease impacts and treatments for single diseases, to the more complex task of modelling the effectiveness of preventative interventions and the interaction of multiple diseases and suites of management practices. In this respect, the gap between scientific information and on-farm practices has also been identified by others (Ivemeyer et al., 2015).

The majority (74.2%) of the dairy specific modelling articles assessed considered a single intervention or no intervention (the latter focusing on risk factors for disease or disease impacts) with only 16 (22.2%) considering more than one intervention. Twenty of the modelling studies focused on disease impacts and risks, 17 on prevention and 25 on treatment interventions. Information about disease impacts can raise farmer awareness about the consequences of a lack of preventative action and allow them to identify the conditions likely to be most damaging (hazard). Studies of risk factors can support farmers in identifying which aspects of their practice and environment are most likely to cause health problems. Combined with estimates of hazard, they can enable the prioritisation of farmer actions to prevent disease. Studies on prevention are then required to support farmers’ choices about the most effective interventions available to tackle the issues identified, including the characterisation of other impacts of change on animal welfare, the environment etc.

Of the 46 modelling studies focusing on a single intervention or no intervention (Supplementary material 3.2), twenty (32.2%) focused on impacts and risks of disease. Only eight models (12.9%) assessed prevention measures, and amongst these four looked only at diagnosis and testing, which may prevent disease outbreaks progressing, but does not in itself control infection or spread. Only one looked at vector control and two at milking timing and conditions. In contrast, ORGSP farmers reported making changes relating to animal stalls and bedding interventions which were not explicitly covered by any model. CONVSP and CONVUK farmers’ changes were diverse, reflected a range of actions not mirrored by attention in modelling. Only CONVUK farmers mentioned vaccination and testing, the latter being addressed by four models. These results suggest that more modelling of practical disease prevention actions (e.g., alterations to stalls and bedding) is required, particularly for ORGSP farmers.

Eighteen modelling studies (29.0%) looking at a single (or no) intervention, focused on disease treatment, with fourteen of these looking at drugs and drug treatment strategies. Such modelling could be useful (along with disease risk and impacts) in raising awareness of, and quantifying, what consequences might follow (e.g., cost of drugs) if prevention measures are not taken by farmers. Drug use may be for prevention as well as for treatment purposes, but this type of preventative intervention was not considered by the farmers surveyed, instead mention of drugs (antibiotics) was associated with wanting to reduce usage. The 16 models considering more than one intervention were equally spread between those considering prevention (nine models–14.5% of all models) and treatment (7 models –11.2% of all models).

Data from the review of models suggest that the input of animal health modelling to farmer decision making is often focused on the quantification of disease risk and treatment costs expressed in economic terms, rather than on the efficacy and impacts of disease prevention actions. Emphasis on the risks of health problems may stimulate a desire to respond protectively, but without adequate information on the impacts and efficacy of potential solutions this may lead to feelings of helplessness and counterproductive responses (Mankad, 2016). Even if disease prevention actions are taken, they may be suboptimal or have unexpected negative consequences if they are carried out with insufficient information and support. The imbalances in modelling support described may be particularly damaging to organic farmers who rely more on disease prevention than treatment, and to farmers who are concerned about non-economic factors as well as productivity when making choices to intervene.

### 3.5 Limitations and next steps

Despite using a range of approaches to distribute the questionnaire to dairy farmers, the number returned (91) was still relatively small. This means that, in some cases, tests may have returned non-significant results due to a lack of statistical power, rather than due to the absence of differences in the population studied. However, qualitative analysis of the free text responses was able to corroborate and provide insights into the quantitative findings, while statistically significant results shed light on important aspects of disease prevention amongst organic and conventional dairy farmers in Spain and the UK. In order to minimize bias in the sample, questionnaires were distributed in the UK and Spain using both electronic and hard copies focused on different settings (such as agricultural shows, knowledge exchange events and online resources for farmers). However, it is recognized that farmers engaged in such settings, and choosing to fill in such a questionnaire, are unlikely to represent harder to reach groups or those with less interest in animal health. Further work is required to explore the perspectives of groups of farmers who are not easily reached by questionnaire-based studies, and to corroborate findings across a larger sample.

## 4 Conclusions

While previous studies have highlighted the requirements of animal health modelling to meet current challenges, this study is the first to directly compare the needs of dairy farmers to the focus and capacity of current animal health modelling in relation to disease prevention. Differences were found between CONVSP and CONVUK dairy farmers and between ORGSP and CONVSP dairy farmers in the factors they reported considering when making changes, and the topics on which they would have liked more information when making a change. ORGSP farmers considered and wanted information on animal welfare most often, while CONVSP and CONVUK farmers considered economic (CONVSP) and practical (CONVUK) factors most often. In relation to information collected on farm, the information used to learn about disease prevention, and how changes were checked for efficacy, the groups of farmers did not differ, gaining information most often from vets and least from the internet, and relying most on their judgement and experience to check the efficacy of changes.

Neither the farmers sampled, nor the models reviewed focused on potential wider impacts of disease prevention measures, such as environmental effects. The findings highlight the importance of improving the modelling of on-farm disease prevention measures to ensure a more holistic approach to animal health, capable of encompassing the multiple outcomes required of a sustainable and resilient livestock sector. Incorporating such wider impacts of health prevention measures into models is likely to require collaboration across modelling disciplines, for example including environmental risk assessment researchers.

The results of this study revealed a strong focus on the economics of animal health in current animal health modelling, in contrast to diverse and system/country specific interests of dairy farmers. Therefore, future animal health modelling and health related outreach and extension should promote a systems-based approach to farming. Models are required which i) are tailored to the needs of specific farming systems and locations, ii) address practical and welfare as well as economic impacts of change, and, iii) when the aim is to share findings directly with farmers, are sensitive to their information delivery preferences. This study has provided specific information for such initiatives, revealing some of the needs and preferences of organic and conventional dairy farmers in Spain and the UK, and highlighting how animal health models might be developed to better meet these needs.

## Funding

This work was supported by the FACCE-JPI knowledge hub Modelling European Agriculture with Climate Change for Food Security (MACSUR) with additional support from INIA (Spain) and BBSRC (UK).

## Ethical statement

The author's state that no animals were used in this study. This article does not contain any sensitive personal data or any other information for ethical review of research.

## Declaration of Competing Interest

The authors declare that they have no known competing financial interests or personal relationships that could have appeared to influence the work reported in this paper. The authors declare the following financial interests/personal relationships which may be considered as potential competing interests: Isabel Blanco-Penedo reports travel was provided by National Institute for Agricultural and Food Research and Technology. Richard P. Kipling reports travel was provided by Biotechnology and Biological Sciences Research Council. This work started with the support of FACCE-JPI knowledge hub Modelling European Agriculture with Climate Change for Food Security

## Appendix Supplementary materials

Image, application 1

Image, application 2

Image, application 3
