# Supplementary material for: Do animal health models meet the needs of organic and conventional dairy farmers in Spain and the UK on disease prevention?
Source: Vet Anim Sci. 2021 Dec 23;15:100226. doi: 10.1016/j.vas.2021.100226 (PMC8718892; doi:10.1016/j.vas.2021.100226)
Supplement: Supplementary file 1 [file mmc1.pdf]

Managing disease risk in dairy herds survey

It is vital that farmers who want to reduce the risk of disease on their farms have access to useful, relevant information

Your answers to this survey will help us understand what types of support can best help you, and other farmers, reduce disease risks. We will use the answers to improve farm advisory and support systems

We don't need your name or address, and any data used will be completely anonymous

1. Where is your farm (country)?

2. What is the main breed in your herd?

3. On average, how many days per year do your cows graze for? (tick one)

241-300	
150-240	
61-149	
1-60	
None	

4. Are you an organic farmer?

YES		NO	

5. Which of these types of health info do you collect on the farm (if any)? (tick all relevant)

**Milk records** (no. cows, individual milk kg, fat/protein, somatic cell count etc.)	
**Breeding records** (date of inseminations, calving interval etc.)	
**Farm book** (deaths, cullings)	
**Other** (please give details)	

6. Where do you get info about animal health and disease risks? (tick all relevant)

Talking to your vet	
Reading farming magazines	
Searching the Internet	
Farm advisors/technical officers/consultants	
Other farmers	
Other (please give details)	

For the next questions, please tell us about choices you made (not things you had to do to meet regulations)

7. What was the biggest change you made on your farm in the last 12 months that you hoped would reduce disease risk (e.g. cleaning the calving pen after each calving)?

(If you haven't made any changes in the last 12 months, please tell us about the last change you made)

8. How did you think this would reduce disease risk (e.g. reduce risk of infections from pen)?

9. Did you think about any of the factors below when you made the change? (tick all relevant)

**PRACTICAL** (e.g. would routines need to change, or would new equipment be needed)		**RISK OF NOT TAKING ACTION** (e.g. likely economic impacts of an outbreak, or effects on farm image)	
**ECONOMIC** (e.g. the cost of change or production impacts)		**WIDER CONCERNS** (e.g. impacts on environment, or alternative options)	
**ANIMAL WELFARE** (if/how the change might affect stress levels etc.)		**OTHER** (please describe)	

10. For the issues you ticked in question 9, can you give details about what you considered?

11. When you were making the change, did you feel you needed to know more about any of these types of issue? (tick all relevant)

**PRACTICAL** (e.g. would routines need to change, or would new equipment be needed)		**RISK OF NOT TAKING ACTION** (e.g. likely economic impacts of an outbreak, or effects on farm image)	
**ECONOMIC** (e.g. the cost of change or production impacts)		**WIDER CONCERNS** (e.g. impacts on environment, or alternative options)	
**ANIMAL WELFARE** (if/how change might affect stress levels etc.)		**OTHER** (please describe)	

12. For the issues you ticked, can you give details of what information would have been useful?

13. What things do you do to see if the change you made was effective? (tick all relevant)

I check monitoring data (e.g. somatic cell counts)		I use practical experience to judge how it is working	
I use info from farm checks (audits etc)		Other (please give details):	
I trial for a while, then decide if it is worth continuing			

14. When you are deciding whether the change you made was effective, would any other kinds of information be helpful? (please describe)

PRIFYSGOL ABERYSTWYTH UNIVERSITY

Thanks for filling in the survey and helping us improve decision support services for dairy farmers

If you want to know more, we would be happy to hear from you. Just email or call: rec21@aber.ac.uk (+441970 823093)
