# Supplementary material for: Do animal health models meet the needs of organic and conventional dairy farmers in Spain and the UK on disease prevention?
Source: Vet Anim Sci. 2021 Dec 23;15:100226. doi: 10.1016/j.vas.2021.100226 (PMC8718892; doi:10.1016/j.vas.2021.100226)
Supplement: Supplementary file 2 [file mmc2.docx]

**Supplementary table 1.** Approach and structured steps used to conduct the systematic search of the literature

| P=Population | TS=(bovine or cattle or cow or cows or heifer* or dairy or calve* or calf*) |
| --- | --- |
| I=Intervention | TS=((organic or extensive* or “farming system” or intensive) NEAR/2 (agricult* or farm* or breed* or "bred" or pasture* or pastor* or system* or rear* or feedstuff or cows or production) or free-range or graz* or roughage or "grass silage" or (housing near/1 system*) or housed or barn* prevent* OR treat*) |
| C=control | TS=( health OR welfare OR disease* OR infection* OR bacteri* OR disorder* OR injurie* OR zoono* OR mortality OR longevity OR pathogen* OR phatologic* OR behavio* OR stereotyp* OR culling* OR metabolic* OR perform* OR producti* OR reproducti* OR fertility OR parasite* OR gastrointestin* OR nematode* OR endoparasit* OR ectoparasit* OR trematode* OR "body condition" OR health management or contaminat* OR virus OR “viral” or ill) AND (model OR “decision support” OR “decision support system” OR “decision support tool” OR “DSS” OR management) |
| O=Outcome | TS=(milk* OR yield* OR perform* OR product* OR physiol OR "metabolic change*" OR "metabolic status" OR lameness OR locomotion OR ketosis OR claw or claws OR hoof or hooves OR "somatic cell count*" OR mastit* OR dystocia* OR "retained placenta" OR "lung worm*" OR pneumonia OR "calving interval*" OR welfare OR days open OR “Reproductive disorders” OR metritis OR endometritis OR “negative energy balance” TB OR BVDV OR fluke OR ‘product*’ OR economic* OR environment* OR emissions, OR sustainab* OR profit* OR infert* |
