# Supplementary material for: Do animal health models meet the needs of organic and conventional dairy farmers in Spain and the UK on disease prevention?
Source: Vet Anim Sci. 2021 Dec 23;15:100226. doi: 10.1016/j.vas.2021.100226 (PMC8718892; doi:10.1016/j.vas.2021.100226)
Supplement: Supplementary file 3 [file mmc3.docx]

**Supplementary Table** **(3.1).** Number of dairy models focusing on each type of health conditions (n=62 scientific papers). **(3.2).** Numbers of models characterizing different aspects of animal health and types of intervention/scenarios within the sample of dairy-specific modelling studies which considered one intervention or no interventions (n=46).

**(1)**

| **Health condition category** | **Number of models** | **Notes on scope** |
| --- | --- | --- |
| Mastitis | 27 | All consider single conditions (mastitis from different causes and different conditions) |
| Gastro-intestinal conditions | 6 | All except one consider single conditions |
| Respiratory conditions | 4 | All consider single conditions |
| Parasite | 7 | All consider one species only |
| Vector control (human disease) | 1 | Focus on control of sand flies |
| Hyperketonemia | 1 | Covers this condition only |
| Foot conditions/lameness | 5 | Three look at more than one foot disorder |
| Fertility | 4 | All look at improving fertility/ reproductive performance in general |
| Pregnancy | 3 | Each model looks at one specific condition |
| Multiple | 4 | Three models focused on general culling decisions and cows´ value. Just one focused on general endemic disease. |

**(2).**

| **Type of Intervention/ Scenario** | **Disease impact/risk** | **Prevention** | **Treatment** |
| --- | --- | --- | --- |
| Drugs (use and comparison of) |  |  | 8 |
| Drug strategy (timing and targeting of drug use) |  |  | 6 |
| Replacement (decision to replace or treat; replacement strategy) |  |  | 4 |
| Vector control |  | 1 |  |
| Impact assessment (of disease) | 11 |  |  |
| Risk assessment (investigation of disease risk factors) | 8 |  |  |
| Immunization (via vaccination or management) |  | 1 |  |
| Diagnosis / testing |  | 4 |  |
| Milking timing/conditions |  | 2 |  |
| Parasite life cycle | 1 |  |  |
